# Number and Size Distribution of Colorectal Adenomas under the Multistage Clonal Expansion Model of Cancer

**DOI:** 10.1371/journal.pcbi.1002213

**Published:** 2011-10-13

**Authors:** Anup Dewanji, Jihyoun Jeon, Rafael Meza, E. Georg Luebeck

**Affiliations:** 1 Applied Statistics Division, Indian Statistical Institute, Kolkata, India; 2 Program in Biostatistics and Biomathematics, Fred Hutchinson Cancer Research Center, Seattle, Washington, United States of America; 3 Department of Epidemiology, University of Michigan, Ann Arbor, Michigan, United States of America; 4 Program in Computational Biology, Fred Hutchinson Cancer Research Center, Seattle, Washington, United States of America; Rice University, United States of America

## Abstract

Colorectal cancer (CRC) is believed to arise from mutant stem cells in colonic crypts that undergo a well-characterized progression involving benign adenoma, the precursor to invasive carcinoma. Although a number of (epi)genetic events have been identified as drivers of this process, little is known about the dynamics involved in the stage-wise progression from the first appearance of an adenoma to its ultimate conversion to malignant cancer. By the time adenomas become endoscopically detectable (i.e., are in the range of 1–2 mm in diameter), adenomas are already comprised of hundreds of thousands of cells and may have been in existence for several years if not decades. Thus, a large fraction of adenomas may actually remain undetected during endoscopic screening and, at least in principle, could give rise to cancer before they are detected. It is therefore of importance to establish what fraction of adenomas is detectable, both as a function of when the colon is screened for neoplasia and as a function of the achievable detection limit. To this end, we have derived mathematical expressions for the detectable adenoma number and size distributions based on a recently developed stochastic model of CRC. Our results and illustrations using these expressions suggest (1) that screening efficacy is critically dependent on the detection threshold and implicit knowledge of the relevant stem cell fraction in adenomas, (2) that a large fraction of non-extinct adenomas remains likely undetected assuming plausible detection thresholds and cell division rates, and (3), under a realistic description of adenoma initiation, growth and progression to CRC, the empirical prevalence of adenomas is likely inflated with lesions that are not on the pathway to cancer.

## Author Summary

The adenomatous polyp (or adenoma) is considered the common precursor lesion for colorectal cancer (CRC). Although the natural history of adenomas is well-characterized in terms of their histopathology and (epi)genomic changes, little is known about their dynamics in the stage-wise progression from the first appearance of an adenoma to its conversion to malignant cancer. By the time adenomas become endoscopically detectable (i.e., are in the range of 1–2 mm in diameter), adenomas are already comprised of hundreds of thousands of cells. A large fraction of adenomas may therefore remain undetected during screening and, in spite of their small (subthreshold) size, could give rise to cancer prior to being detected. It is therefore of importance to establish what fraction of adenomas is detectable, both as a function of the age at screening for colorectal neoplasia and the size (threshold) above which adenomas can be detected reliably. Here we derive mathematical expressions for the distribution of adenoma number and sizes based on a recently developed stochastic model for CRC, which has previously been calibrated and validated against age-specific CRC incidence data.

## Introduction

Adenomatous polyps (or adenomas) in the large intestine are considered benign precursors of colorectal cancer (CRC) and both clinical and molecular evidence suggest that they may sojourn for many years before turning into cancer [1], [2]. For this reason, adenomas are considered a primary intervention target if detected and removed before they become malignant. However, questions remain regarding the significance of their histopathology, molecular signatures, as well as their number and sizes in average risk individuals. Since endoscopic screening for neoplastic lesions is generally limited by macroscopic detection thresholds (of the order of a few mm in caliper size), a large fraction of adenomas may actually be missed, especially if the bulk of adenomas is too small for detection. Potentially, such “occult” adenomas could give rise to cancer before they are detected by endoscopy. Here we use a biologically-based model of colorectal carcinogenesis, which has previously been fitted to the age-specific incidence of CRC, to compute the number and size distributions of adenomas. Of particular interest is the fraction of detectable adenomas, as functions of age, detection threshold and the underlying cell kinetics in the adenomas.

The underlying multistage clonal expansion (MSCE) model for CRC upon which our results are based explicitly considers the initiation, promotion and malignant conversion of adenomas [3]–[8]. According to this model, adenomas arise from normal colonic stem cells that suffer at least two rare rate-limiting events. We interpret these events as the biallelic inactivation of a tumor suppressor gene, in particular the APC tumor suppressor gene, which is the gene responsible for familial adenomatous polyposis (FAP), and which is frequently mutated in colorectal neoplasia [9]. The inactivation of APC is understood to occur in colonic crypts (the fundamental proliferative unit in the colon) whose stem cells have previously acquired a mutation at one of the two APC alleles. Because the process of adenoma formation may involve additional genes (such as KRAS), we extend the model framework to accommodate additional rate-limiting mutations for the initiation of an adenoma and generalize the mathematical derivation of their number and size distribution accordingly. However, there is both clinical and experimental evidence that the number of requisite rate-limiting events or mutations for adenoma initiation is small. Once a stem cell is initiated in this model, it is free to proliferate. The basic version of our CRC model assumes that adenoma initiation occurs when the remaining wild-type copy of the APC tumor suppressor gene is deleted or mutated in a stem cell of a (pre-initiated) APC+/− colonic crypt. In a more realistic model, which is supported by recent experimental findings in the murine system [10], we also model the transient amplification of APC−/− stem cells prior to their clonal expansion, effectively adding a stage to the initiation process [10]–[12].

The theoretical results derived here are complemented by model predictions for the adenoma size distributions and their (age-specific) prevalence based on parameter estimates obtained previously from fitting cancer incidence data. Since not all biological model parameters can be directly estimated from incidence data alone (non-identifiability issue), we explore the sensitivity of our findings by varying unknown parameters, such as the cell division rate of initiated stem cells, within their plausible ranges. In spite of the model uncertainties and the lack of precise clinical data on adenoma number and sizes, a biologically based approach that is broadly consistent with the pathogenesis of CRC makes it possible to explore more rationally the impact of risk factors and interventions on adenoma development and cancer progression.

## Model

### The Multistage Clonal Expansion (MSCE) Model

First we briefly review the MSCE model for CRC and then introduce the notation for the relevant stochastic processes involved in the formation of adenomas. We have previously derived expressions for the number and size distribution of non-extinct pre-malignant clones in the context of the two-stage clonal expansion (TSCE) model [13]. This model assumes that the clones develop from a (deterministic) source of progenitor cells via a non-homogeneous Poisson process. An important extension of this work was put forward by Dewanji et al. [14] for the size distribution of a random sum of Poisson-generated (pre-malignant) clones, which corresponds to a generalized Luria-Delbrück (GLD) distribution for mutant colonies. A hallmark of this distribution is a long tail reflecting large fluctuations of the total (mutant) population size. A further extension derived expressions for the number and size distributions of pre-malignant clones conditioning on observations from individuals who have not previously been diagnosed with CRC [6].

#### Adenoma development

**Figure 1:**
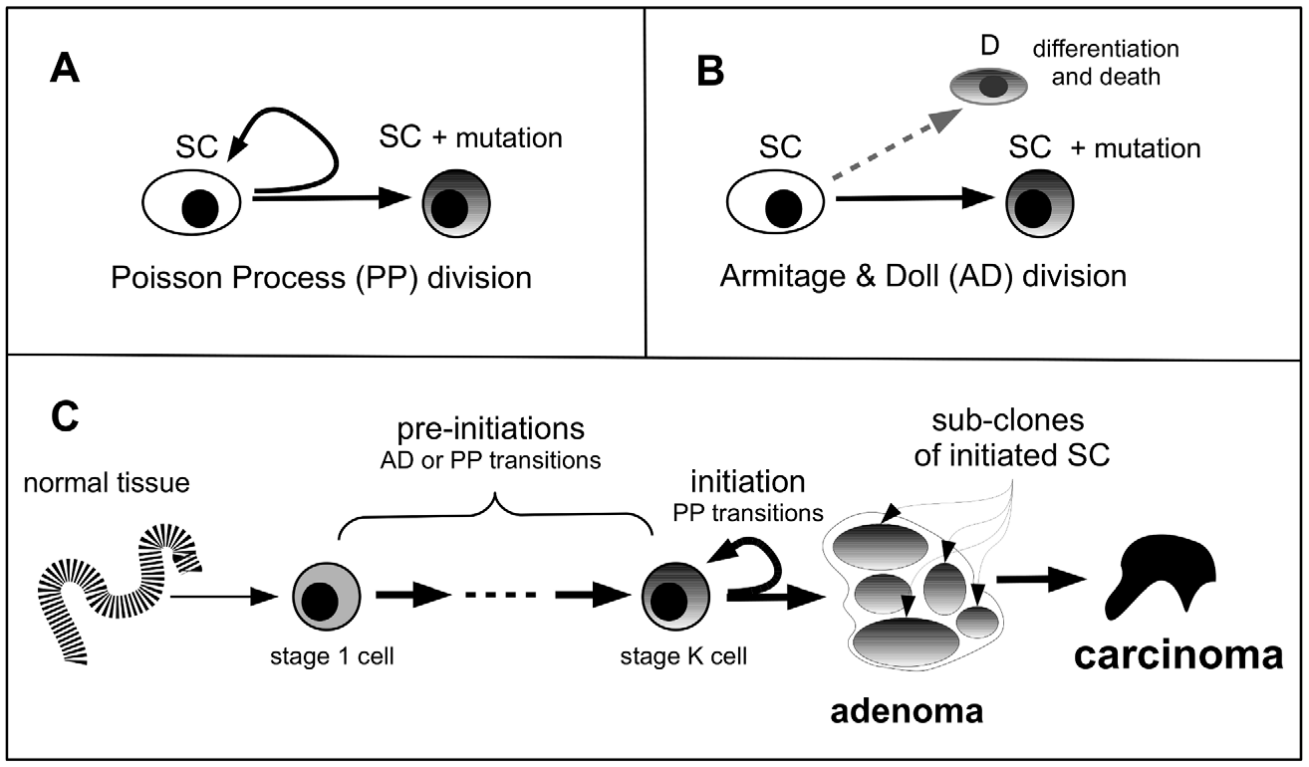
Multistage clonal expansion (MSCE) model. SC: stem cell, D: differentiated cell.

For colorectal adenomas, the MSCE model assumes that adenomas arise within colonic crypts maintained by immortal stem cells that have accumulated 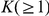 requisite (epi)genetic pre-initiation events. In this model, adenomas are allowed to be multi-focal since the initiation process, starting from the time when the 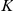
th pre-initiation event has occurred in a stem cell, is a point process representing the continuous generation of initiated cells from the 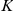-stage cell, the progeny of which is free to undergo clonal expansion. In this picture, pre-initiated stem cells are blocked from clonal expansion; however, they are allowed to divide asymmetrically to generate daughter cells that will (ultimately) undergo terminal differentiation (see Figure 1). This is described in more detail below. To give an example, for a 3-step initiation model (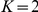), adenomas arise from crypts whose stem cell population sustains two consecutive hits (e.g., the inactivation of both alleles of the APC gene). In this case initiating events occur when the crypt stem cells suffer a third event.

#### Pre-initiation events

There are two distinct biological ways for pre-initiation events to occur at the cellular level. A pre-initiation event may occur in a stage 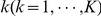 stem cell via an asymmetric stem cell division in which a mutation occurs in one of the two daughter stem cells. In other words, a stem cell in the 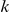
th pre-initiation stage may divide asymmetrically to yield two daughter stem cells, one in stage 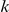 and the other in stage 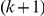 which acquires a new mutation (Figure 1A). This process is mathematically modeled as a Poisson process with intensity rate 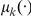. In this case, both daughters are retained in the stem cell compartment. The other possibility is represented by another kind of asymmetric stem cell division which causes one daughter stem cell to acquire a mutation leading to a transition of this cell to stage (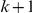), while the other daughter is committed to differentiation (Figure 1. B). For historical reasons, we refer to this event as an Armitage-Doll (AD) type transition [15].

For both cases we assume that a stage 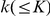 stem cell is not yet initiated and therefore lacks the potential for clonal expansion via symmetric cell divisions. However, once a stem cell enters stage (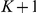), it is considered initiated and free to undergo clonal expansion. Furthermore, all stem cells in the pre-initiation stages are assumed immortal.

#### Multi-focal nature of an adenoma

In the context of the MSCE model for CRC, an adenoma consists of the collection of all initiated (stage 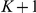) cells that derive from a single stage 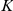 progenitor cell. This definition, while not unique, is consistent with the assumption that the stage 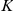 progenitor may be subject to transient amplification, which is represented by frequent Poisson ‘emissions’ of initiated (stage 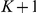) cells that are free to undergo independent clonal expansions resulting in the formation of multiple sub-clones (Figure 1C).

We call this ensemble of sub-clones an *adenoma* or *adenomatous polyp*. Since information on adenoma number and sizes is typically obtained via screening of individuals who have not previously been diagnosed with CRC, we will also derive the results of the model conditioning on no prior clinical detection of CRC. For this purpose, we assume that the last rate-limiting event (with rate 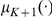) in the MSCE model, which is usually associated with the malignant conversion of an initiated cell, represents detection of a clinical cancer as in Jeon et al. [6].

#### Basic notation

Suppose there are 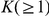 pre-initiation stages. We refer to the 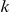
th pre-initiation event as a 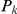-mutation and the cells, which have gone through this 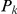-mutation, as 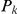-cells (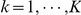). For completeness, we refer to the normal stem cells as 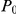-cells. The generation of 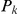-cells from one 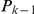-cell can be modeled through a non-homogeneous PP with rate 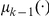 per cell. Alternatively, it can be modeled as a direct transition of a 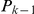-cell into a 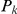-cell, the AD-type transition referred to above, with hazard rate 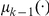 and density 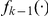 for the waiting time of a 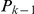-cell before the 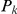-mutation takes place. For the AD type transition, it is assumed that the functions 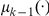 and 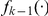 depend only on the time since the 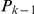-mutation occurred. If 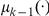 is a constant, then 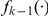 is the density of an exponential distribution. Once a 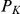-cell is formed (by means of a 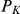-mutation), it generates initiated cells (i.e., 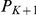-cells) according to a non-homogeneous PP with rate 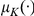 and the initiated cells grow according to a linear birth and death process with rates 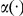 and 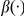, respectively. Note, the initiation of 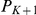-cells and their clonal expansion recapitulates the two-stage clonal expansion (TSCE) model for which explicit solutions for the number and size distributions have been derived [13]. Figure 1.C illustrates the MSCE model for carcinogenesis. The pre-initiation events can be either PP-type or AD-type transitions. However, the first step in the MSCE model represents the successive (random) generation of 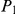 mutant stem cells over time and throughout the normal tissue, which is assumed to be very large in size - about 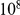 normal stem cells in colon and rectum combined. Hence, without loss of generality, the MSCE model assumes that the arrivals of the first mutations are of the PP-type.

#### Size and detection of an adenoma

Suppose we observe, for an individual at a particular time 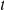, the number of detectable adenomas, 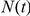, and their sizes (in terms of the number of initiated or 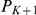-cells in each adenoma), 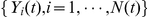. We assume that an adenoma is detectable with probability one if its size is greater than a fixed threshold 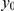. In the following sections, we derive the joint distribution of 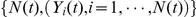 for different values of 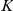 and different assumptions regarding the type of pre-initiation process (i.e., PP or AD). For 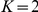, we essentially consider the model recommended by Luebeck & Moolgavkar [12] for CRC and used by Jeon et al. [6] for evaluating screening strategies for adenomas in colon and rectum. The latter study provided an efficient approach to simulate the natural history of CRC by recognizing that the size of the adenomas, given the arrival time of a 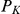-cell, follows a GLD distribution as derived by Dewanji et al. [14].

Let 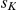 be the time of a particular 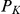-mutation, that is, the arrival time for a progenitor cell (a 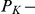
cell) for an adenoma. Then this progenitor cell will generate initiated cells (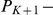
cells) by a Poisson process, and each initiated cell forms a sub-clone of 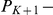
cells via a clonal expansion. Note, 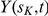 is the (random) sum of all sub-clones of initiated cells (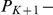
cells) that were generated from the 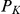 progenitor stem cell which was born at time 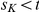.

In particular, when the initiation rate 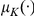, the cell division rate 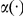, and the cell death rate 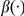 are constants, the GLD distribution reduces to a Negative Binomial distribution (Eq. (3.34) in [6]), given by
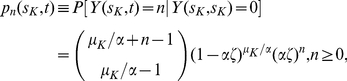
(1)where 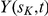 denotes the size of the adenoma at time 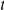, given the time of 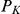-mutation 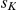, and
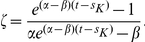
Under these assumptions, the sub-clones that lead to this (multi-focal) distribution arise from initiated 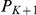-cells that expand clonally by following a linear birth-death process. For this reason, the results derived here are readily applied to the situation when the sub-clones are also identified clinically. Conditioning this distribution on adenomas that occur in cancer-free individuals, i.e., individuals who have not had a prior occurrence of CRC, we have (see Eq.(3.30) in [6])
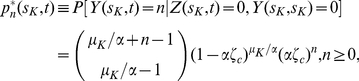
(2)where
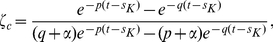
(3)

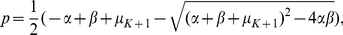
(4)

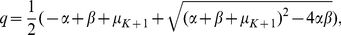
(5)and 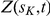 is the indicator variable for clinical detection of cancer at time 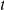 with 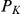-cells born at time 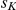.

### Number and Size Distribution for 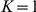


For 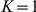 we have only one pre-initiation event, defined by a 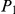-mutation, and 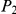-cells are the initiated cells. As mentioned in the previous section, the 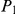-mutation follows a PP formulation. Let 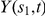 denote the size of the adenoma at time 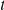 with the first pre-initiation (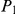-mutation) time 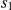. The distribution of 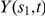 is given by the GLD distribution previously derived by Dewanji et al. [14], for the process originating at time 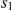 and involving the initiation rate 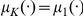 and the birth and death rates of the initiated cells given by 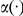 and 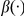, respectively.

As mentioned before, we assume that the generation of 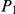-cells follows a non-homogeneous PP with rate 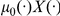, where 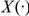 gives the deterministic growth curve for the normal stem cells in the tissue. Let 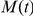 be the number of first pre-initiations (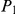-mutations) by time 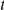 and let 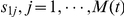 be the occurrence times of these 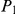-mutations. Also, write 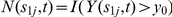, where 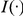 is the indicator function. That is, 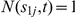, if the corresponding adenoma is detectable at time 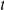, and 0 otherwise. Then, the number of detectable adenoma 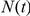 can be written as a filtered Poisson process
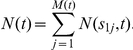
The probability generating function (PGF) of 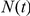 can be written as
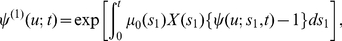
(6)where 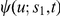 is the PGF of the binary variate 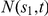 with success probability
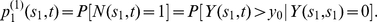
Note that 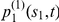 is the probability that a 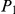-mutation taking place at time 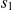 results in a detectable adenoma at time 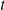. This probability can be obtained from the distribution of 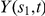. For constant parameters, this reduces to, using (1) with 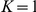, 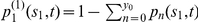.

#### Adenoma prevalence

It follows that the number of 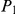-cells which lead to detectable adenomas at time 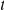 follows a non-homogeneous PP with rate 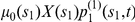, with 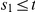, and 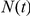 has a Poisson distribution with mean 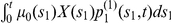. Since the adenoma prevalence is defined as the probability of at least one detectable adenoma at age t, it is given by
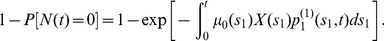
(7)


#### Detection probability and size distribution of adenomas

The probability of detecting an adenoma of size 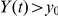 at age 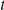 is given by
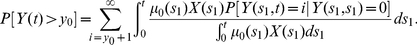
(8)For constant parameters, this reduces to 
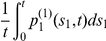
. Similarly, the size distribution of a detectable adenoma at age 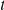 is given by
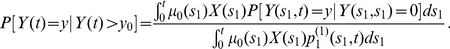
(9)


#### Likelihood for the number and size of detectable adenomas

Using the properties described above, it is straightforward to show that the joint probability (or likelihood 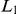) of having 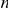 detectable adenomas with sizes 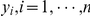 at age 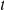, i.e, 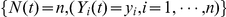 is
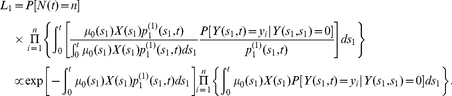
(10)


#### Extension to observations in individuals with no prior CRC

Here we derive analogous results for cancer-free individuals, i.e., individuals who have not developed CRC by time 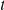. To this end, we require the conditional probability 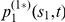 that a 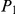-mutation taking place at time 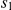, results in a detectable adenoma at time 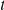 prior to developing CRC. Hence, we need to compute the conditional probability 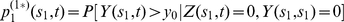. For constant parameters, using (2), 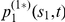 can be calculated as 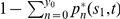.

#### Adenoma prevalence among individuals with no prior CRC

As before, the number of detectable adenoma at time 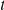 in a cancer-free individual, 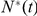, follows a Poisson distribution with mean given by 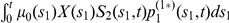, where the two-stage survival function 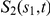 represents the probability that a 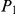-cell born at time 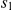 does not lead to CRC by time 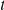. Thus, the adenoma prevalence conditioned on cancer-free is given by
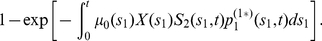
(11)


For constant parameters, this two-stage survival function has been derived previously, i.e.
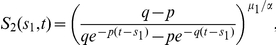
where 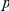 and 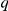 are defined in (4) and (5) with 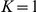 (see [6], [12] for details).

#### Detection probability, size distribution, and likelihood function for adenomas in individuals with no prior CRC

Here we provide analogous expressions for the detection probability (8) and size distribution (9), but properly conditioned on no occurrence of prior CRC. The probability of detecting an adenoma at age 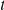 with size 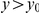, conditioned on no prior CRC, can be written as
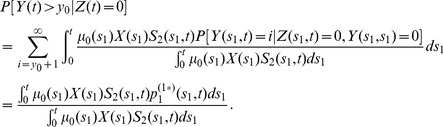
(12)


Similarly, for the size distribution of detectable adenomas (i.e., their sizes exceeding the threshold 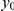) at age 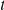, conditioned on no prior CRC, we find
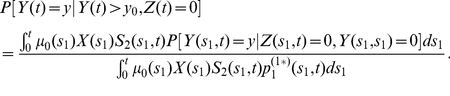
(13)


Finally, following the derivation of (10), the joint distribution of the number and sizes of detectable adenomas, in a cancer-free individual, can be written as
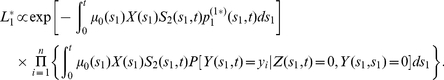
(14)


### Number and Size Distribution for 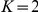


Here the two pre-initiation events (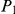
- and 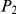-mutations) precede initiation and growth of initiated 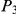-cells into sub-clones. Let 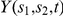 denote the size of the adenoma at time 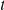 with the corresponding 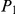
- and 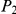-mutations taking place at times 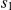 and 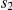, respectively. Note that the distribution of 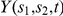 is given by the GLD distribution originating at time 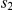 and involving the initiation rate 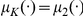 and the birth and death rates of the initiated or 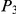-cells, given by 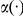 and 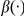, respectively. We derive explicit expressions for the number and size distributions for the case when both 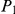
- and 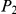-mutations are of PP-type. The case when the 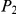-mutations are of AD-type is described in the online supplement (Text S1).

As before, the number of detectable adenomas at time 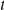 can be written as
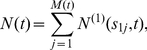
(15)where 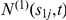 is the number of detectable adenomas that emerged from a 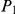-cell born at time 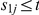. Then, as in (6), the PGF of 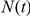 can be expressed by
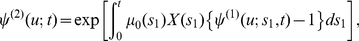
(16)where 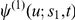 is the PGF of 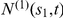. Using the Lemma and eq. (10) of Dewanji et al. [14], we have further
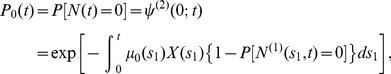
(17)and for 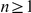,
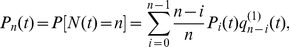
(18)where, for 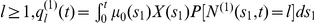.

Again,
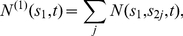
(19)where this sum is over all the 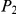-mutations by time 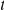 that occurred at times 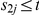 and which emerged from a 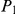-cell that was born at time 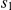. Here, 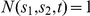 if the adenoma originated from the 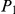-cell born at time 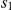 and the 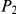-cell born at time 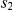 is detectable at time 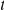 and 0 otherwise; that is, 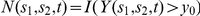. Note that 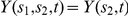, since 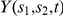 does not depend on 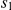. Therefore, as in the previous section, 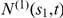 is a filtered PP with the PGF 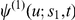, similar to that in (6). Also, 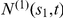 is a non-homogeneous PP with rate 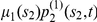, for 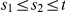, and for fixed 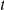, is a Poisson variate with mean 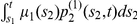, where
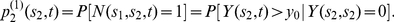
(20)This probability can be obtained from the distribution of 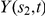 given in (1).

#### Adenoma prevalence


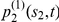
 has the same form as that of 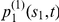 but with 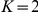. Thus, using (17), the adenoma prevalence is calculated by
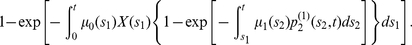
(21)The distribution of 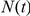 can now be obtained by using (18), and the expected number of detectable adenomas can be readily obtained using (16), i.e.
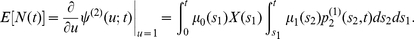
(22)


#### Detection probability and size distribution of adenomas

The probability of detecting an adenoma of size 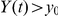 at age 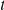 is given by
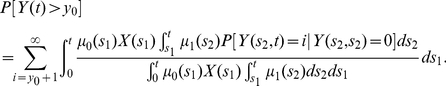
(23)


For constant pre-initiation rates 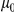 and 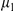, and a constant normal stem cell number 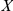, this reduces to 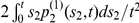. Similarly, the size distribution of a detectable adenoma at age 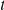 is simply given by
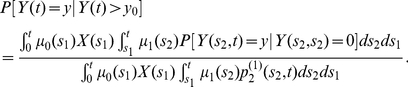
(24)


#### Likelihood for the number and size of detectable adenomas

Let 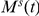 be the number of ‘special’ 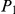-mutations that lead to at least one detectable adenoma at time 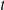. Because of the filtered-Poisson-process nature of the generation of the adenomas, the occurrence of such special 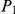-mutations follows a PP with rate 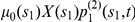, for 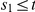, where 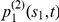 is the probability that a 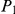-mutation that occurred at time 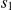 leads to at least one detectable adenoma by time 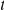. Using the distribution of 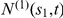, we have
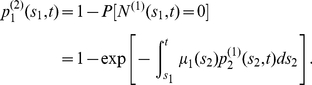
(25)


We now turn to the joint distribution of having 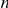 detectable adenomas with sizes 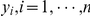 at age 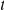, i.e, 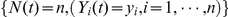, when 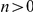. First, let 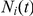 denote the number of detectable adenomas arising out of the 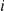
th ‘special’ 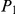-mutation with sizes 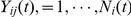. Clearly, 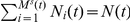. Then, given 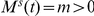, the events 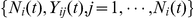, for 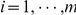, are independent and identically distributed with
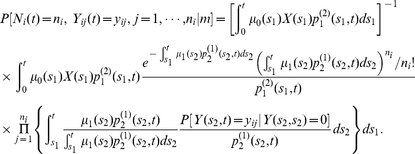
(26)Therefore, the joint probability of 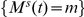 and 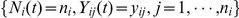 for 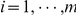, is given by
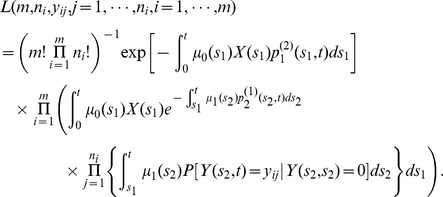
(27)


In order to derive the joint distribution of 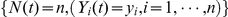, using (27), let 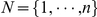 denote the set of labels for the 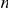 clones. Then, summing over all possible 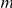 and the possible partitions of 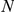 with 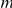 subsets, the likelihood is given by
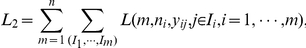
where 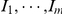 are disjoint subsets of 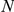 with 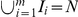, and the second sum is over all such 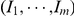 with 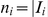, the number of labels in 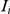. If 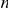 is not very large, this sum is not difficult to work with.

#### Extension to observations in individuals with no prior CRC

Analogous results can be obtained for observations in individuals who have not developed clinical CRC by time 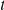 by directly conditioning the Poisson rates and using conditional size distributions for 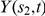. For example, the relevant joint distribution of adenoma number and sizes (i.e., (27)) can be obtained by the following substitutions:
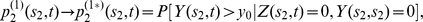


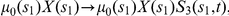


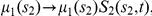
and 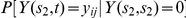 is replaced by
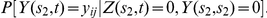
Note, 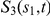 and 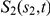 represent the respective probabilities that a 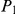-cell born at time 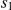 and a 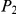-cell born at time 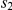 do not give rise to CRC by time 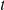. In other words, they are the tumor survival functions of a 3-stage and 2-stage carcinogenesis model, respectively. For constant parameters, these survival functions are as following (see [6], [12] for the derivation):
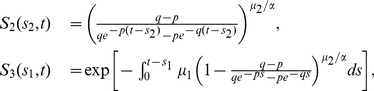
(28)where 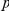 and 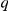 are defined in (4) and (5) with 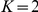 (see [6], [12] for details). Thus, the conditional expression for adenoma prevalence is given by
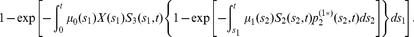
(29)


The expected number of detectable adenomas conditioned on no prior CRC can be obtained through analogous replacements in (22).

#### Detection probability and size distribution for adenomas in individuals with no prior CRC

As before for the case with 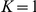, we also provide analogous expressions for the detection probability (23) and size distribution (24), but properly conditioned on no previous occurrence of CRC. The probability of detecting an adenoma at age 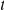 with size 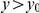, becomes
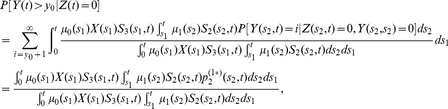
(30)and for the size distribution of detectable adenomas (i.e., their sizes exceeding the threshold 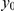) at age 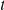, conditioned on no prior CRC, we have
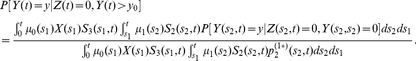
(31)


### Number and Size Distribution for General 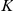


The notation introduced in the previous section is easily generalized to 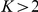. The random variable 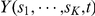 denotes the size of the adenoma at time 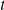 with the corresponding 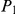-, 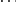,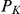-mutation times 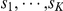, respectively. The distribution of 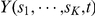 is, as before, given by the GLD distribution with time origin at 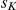 and with initiation rate 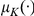. Initiated 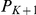-cells divide or die with rates 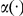 and 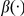, respectively. Importantly, 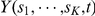 depends on 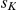 alone (i.e., 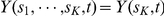), and the distribution is given by (1) for constant parameters.

Various combinations of AD-type and PP-type generations for the 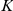 pre-initiations are possible, but the formulation of the likelihood becomes more complicated. The special cases when all pre-initiations are of PP-type or AD-type are covered in the online supplement (Text S1).

## Results

The derived expressions allow us to readily predict both observable and unobservable numbers of pre-malignant tumors in a tissue. Such predictions are helpful in validating cancer models using intermediate endpoints on precursor lesions, in particular their number and sizes. Furthermore, being able to predict the unobserved portion of precursor lesions is of clinical relevance for early detection and cancer prevention. Here, we illustrate the utility of the derived expressions using the example of colonic adenomas. Specifically, we present the predicted size distribution of adenomas and the age-specific adenoma prevalence, i.e., the probability of finding at least one observable adenoma in an individual as a function of age. Since population-level screening is typically performed on asymptomatic individuals, we also condition on individuals having not developed cancer in the tissue of interest at the time of observation.

The predictions presented here are for 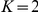 as described above. The underlying CRC model for cancer incidence is the 4-stage model previously derived by Luebeck & Moolgavkar [12] and updated by Meza et al. [7]. The alternative model (PP for 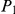
- and AD for 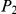-mutation for 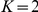 in the online supplement (Text S1)) yields very similar results (not shown). Importantly, not all biological parameters of the MSCE/CRC model are estimable from incidence data alone. For example, for the 4-stage model used here, only the parameters 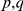, the product (slope parameter) 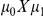, and the ratio 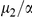 are identifiable. However, if the cell division rate of initiated cells, 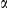, is known, all parameters of the model can be determined (assuming that the number of normal tissue stem cells, 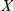, is known and that 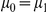). For our illustrations, we choose plausible values for the cell division rate 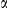, but keep the values of 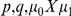, and 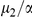 as estimated by Meza et al. [7]. This affords explicit computation of the adenoma number and size distribution without altering the fits of the model to the observed CRC incidence.

**Figure 2:**
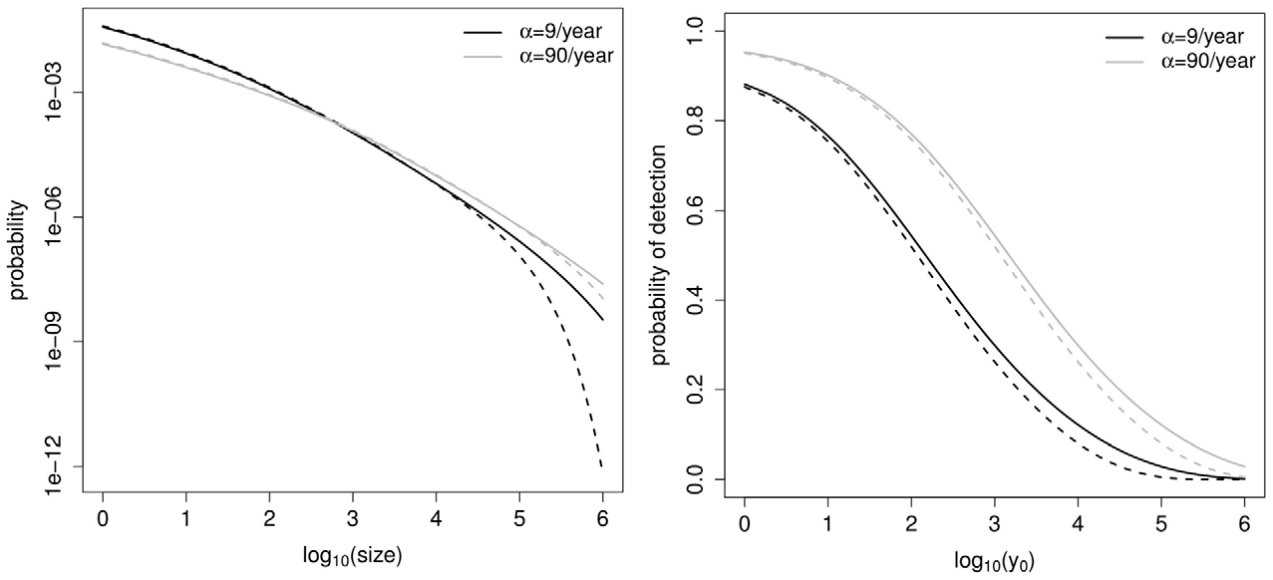
Size distribution of a detectable adenoma and probability of detection of an adenoma. Left panel: predicted unconditional (solid) and conditional (dashed) size distribution of a detectable adenoma at age 70 using the parameter estimates obtained by [7] for females in SEER with 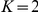 and 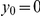. The cell division rate of initiated cells, 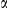, is assumed as either 9 or 90/year. Right panel: the probability of detection of an adenoma at age 70 as a function of the detection threshold 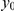. Otherwise same as left panel.

Figure 2 (left panel) shows the predicted size distribution of non-extinct adenoma without an imposed detection threshold (i.e., 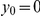) for the model with 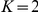. With constant parameters, both the unconditional and conditional (on no prior CRC development) size distributions of detectable adenoma are given by expressions (24) and (31), respectively.

For sizes sufficiently large, the unconditional adenoma size distribution is roughly log-log-linear, while the conditional size distribution shows departures from this behavior for sizes above 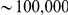 cells, i.e., when the risk of an adenoma-to-carcinoma transition increases more rapidly. This phenomenon is more noticeable when the cell division rate 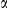 is lower. Figure 2 (right panel) shows the probability of detecting an adenoma at age 70 as a function of the detection threshold 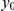 for both unconditional (solid) and conditional (dashed) adenoma size distributions. Higher cell division rates (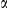) give rise to larger adenoma sizes and hence lead to higher detection probabilities even though the net cell proliferation rate (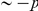) is approximately the same. For constant parameters, the unconditional and conditional detection probabilities are given by (23) and (30), respectively. This figure reveals that even for relatively small (i.e., sensitive) thresholds of a few thousand cells, many adenomas may go undetected. However, the precise proportion of detectable adenomas depends on the cell division rate 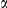 with higher values of 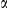 making detection more likely.

**Figure 3:**
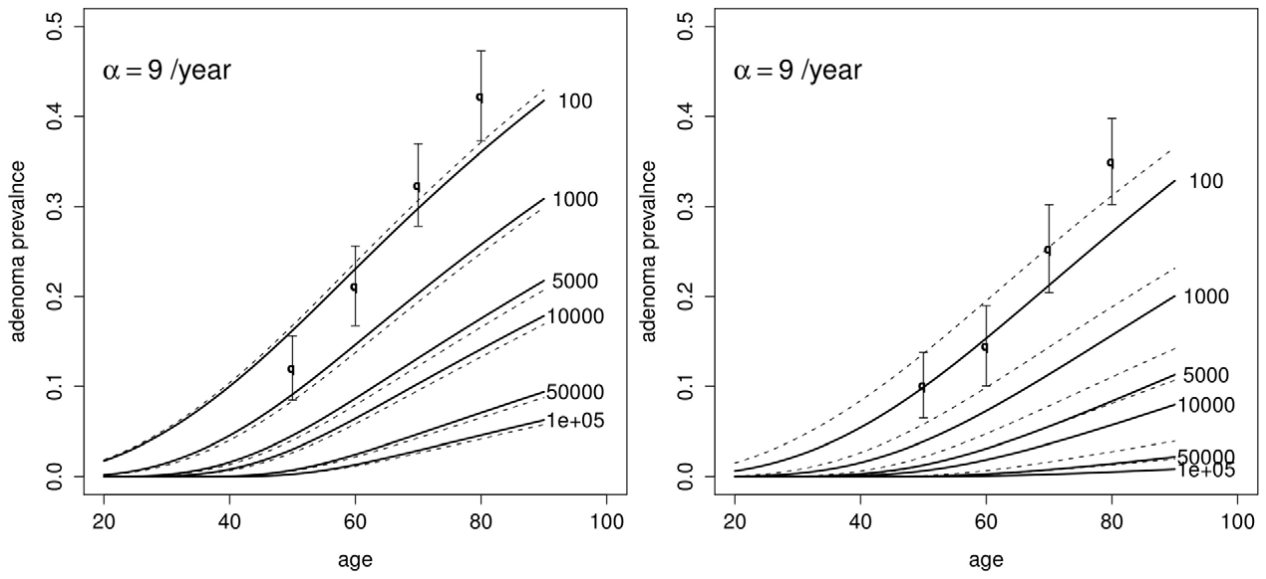
Adenoma prevalence. Predicted adenoma prevalence for both males (Left panel) and females (Right panel) as a function of age and various observation thresholds 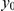 using the models with 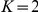 (solid lines) and 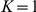 (dashed lines). Empirical data from Clark et al. [16] in filled circles.

Figure 3 shows the predicted age-specific adenoma prevalence in asymptomatic individuals for both males and females and for the models with 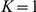 and 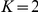, as described by Meza et al. [7], including their dependence on the observation threshold 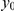. The prevalence is defined as the probability of at least one detectable adenoma at age 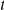, and is given by (11) for 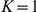 and (29) for 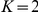. In comparison with careful observations from autopsy studies [16], the model under-estimates these empirical data (represented by filled circles) unless one accepts a very small number of initiated stem cells to be observable. There are several explanations why the model-generated expected prevalence of adenoma might be lower than the clinical data would indicate (see the end of Discussion).

## Discussion

We have previously derived number and size distributions of pre-malignant clones in the context of the two-stage clonal expansion (TSCE) model of carcinogenesis [13], [17] and more recently established a formal connection of these results with fluctuation analyses based on the Luria-Delbrück distribution [14]. The mathematical tools derived in these papers were subsequently applied to the problem of screening for colorectal adenoma allowing for interventions resulting from their complete or incomplete removal [6]. These explorations, however, required time consuming computer simulations. In contrast, here we derive mathematical expressions that allow us to readily compute adenoma number and size distributions without simulation. These expressions can form the basis for computing the likelihood of adenoma data from screening studies involving sigmoidoscopies, colonoscopies and computed-tomographic colonographies, and thus are of significantly practical importance. Moreover the analytical form of the likelihood function allows for parameter estimation and likelihood-based hypothesis testing. Analyses of such data will be forthcoming and are the subject of a separate paper.

Our previous analyses of CRC incidence data suggest that 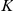, the number of requisite pre-initiation mutations, is indeed small [7], [12]. 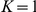 corresponds to a ‘two-hit’ model for initiation, in essence representing the biallelic inactivation of a tumor suppressor gene (Knudson's recessive oncogenesis) [7]. A model with 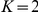 may describe both the inactivation of a tumor suppressor gene as well as the activation of an oncogene [7], [12]. Here, we also treat the case of general 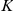, which can be viewed as a model for clonal evolution due to the tree-structure where the nodes represent immortal mutant stem cells that will give rise to specific sub-clones which may or may not be identified as such. We distinguish between two types of rate-limiting events, one that generates (potentially multiple) mutations via asymmetric cell division while preserving the progenitor stem cell from which the mutations arose (PP-type), and one that leads to a transition of a progenitor cell into one cell that acquires a new mutation (AD-type). The MSCE model used here assumes that all events that lead to initiated cells are PP-type. This is only a mild restriction since for rare events the PP-type emission is equivalent to a AD-type transition (see Figure 1). For frequent (high rate) events, the AD-type transition looses its rate-limiting nature and can be ignored, while the high rate (PP-type) process leads to the accumulation of multiple clones and thus has the potential to capture non-mutational events, such as the transient amplification of proliferative cells from resident stem cells in the colonic crypts. Once a stem cell is considered initiated, i.e., is of type 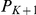, we assume that it undergoes a stochastic birth-death process. This leads to the GLD distribution introduced in [14] for the adenoma size 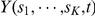, which reduces to a Negative Binomial distribution for constant parameters. Note, however, our formalism is more general and can accommodate other growth models that do not result in a GLD size distribution for the initiated cell population emerging from a 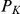 progenitor cell [17], [18].

Finally, we wish to comment on the predictions of the model for the age-specific adenoma prevalence (Figure 3). In comparison with the empirical data, our predictions appear too low. However, the discrepancy depends on what is assumed for the initiated stem cell division rate 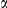 and the detection threshold 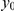. While the range of plausible values for 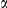 is limited by how fast initiated stem cell can cycle in the adenoma (unlikely more than 2–3 times a week), it is not clear what fraction of cells in an adenoma is truly at risk for malignant transformation [10]. Assuming that a 1 mm adenoma, the caliper size detection limit cited by Clark et al. [16], contains about 500,000 cells [19] and that only 1–10% of cells in an adenoma are tumor stem cells [10], [20], 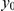 may be as low as 5000 cells and therefore the discrepancy may be less dramatic. Alternatively, one might include pre-initiated cells in the adenoma size count. However, our assumption is that pre-initiated cells do not expand clonally, although they may increase in number as a result of multiple births of the same type of mutation from a single stem cell over time (via Poisson process emissions). Thus, since locus-specific mutations are rare (of the order of 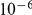 to 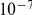 per year), the contribution of pre-initiated cells to the overall number of cells in an adenoma is likely very small.

It is well-recognized that adenomas can be genetically diverse and differ widely in their neoplastic potential. Indeed, adenomas have been suggested to regress implying that there are adenomas that are not on the pathway to cancer [21], although regression may simply reflect the stochastic nature of tumor growth. A more intriguing possibility of resolving the discrepancy is that adenomas go through a growth-bottleneck (i.e., stagnancy) before they can become cancerous. In this scenario, adenomas might sojourn in a reservoir until an activating mutation or change in tumor microenvironment releases them from arrest [22], [23]. Although incorporating this scenario into the MSCE model may be challenging, the framework presented here is independent of the particular dynamics of the initiated cells and the number of clonal expansions assumed.

## Supporting Information

Text S1 Supplementary methods and results. A tabular glossary which summarizes our notation and succinctly defines the model parameters and terminology in use.
(PDF)
