# Supplementary material for: Number and Size Distribution of Colorectal Adenomas under the Multistage Clonal Expansion Model of Cancer
Source: PLoS Comput Biol. 2011 Oct 13;7(10):e1002213. doi: 10.1371/journal.pcbi.1002213 (PMC3192823; doi:10.1371/journal.pcbi.1002213)
Supplement: Text S1 — Supplementary methods and results. A tabular glossary which summarizes our notation and succinctly defines the model parameters and terminology in use. (PDF) [file pcbi.1002213.s001.pdf]

Number and Size Distribution of Colorectal Adenomas under the Multistage Clonal Expansion Model of Cancer: Supplementary Material

Anup Dewanji, Jihyoun Jeon, Rafael Meza, E. Georg Luebeck

This supplement covers special cases that were not explicitly treated in the main text (e.g., replacing PP-type pre-initiation steps with AD-type steps) and the generalization to models with more than two pre-initiation steps, i.e., $K > 2$. In addition, we introduce a tabular glossary (Table 1 in Text S1) which summarize our notation and succinctly define the model parameters and terminology in use.

Number and Size Distribution of detectable adenomas for $K = 2$

PP for P_1- and AD for P_2-mutation for $K = 2$

For $K = 2$, there are two pre-initiation events (P_1- and P_2-mutations) before initiation occurs. In the main text, we have described a modeling scenario, in which both P_1- and P_2-mutations are PP.

In many situations, the second pre-initiation (that is, the P_2-mutation) appears to be rare so that, in practice, there can be at most one such event from each P_1-cell. Then, a AD-type transition for the P_2-mutation is an alternative, which simplifies the results considerably. Each P_1-cell, in this modeling, has a random waiting time with density $f_1(\cdot)$, before converting into a P_2-cell, which then becomes a detectable adenoma at time t with probability $p_2^{(1)}(s_2, t)$ (See the section 'Number and Size Distribution for $K = 2$' in the main text). Therefore, a P_1-cell born at time s_1 has the probability

$$p_1^{(AD)}(s_1, t) = \int_{s_1}^{t} f_1(s_2 - s_1) p_2^{(1)}(s_2, t) ds_2$$

to become detectable at time t.

Adenoma Prevalence: The number of detectable adenomas $N(t)$ can, therefore, be written as a filtered PP, as in the section 'Number and Size Distribution for $K = 1$' in the main text, with $p_1^{(1)}(s_1, t)$ replaced by $p_1^{(AD)}(s_1, t)$. Consequently, the generation of P_1-cells, which lead to detectable adenomas at time t, follows a non-homogeneous PP with rate $\mu_0(s_1)X(s_1)p_1^{(AD)}(s_1, t)$, for $s_1 \le t$, and $N(t)$ follows a Poisson distribution with mean $\int_0^t \mu_0(s_1)X(s_1)p_1^{(AD)}(s_1, t)ds_1$. Thus, the adenoma prevalence is given by

$$1 - \exp\left[- \int_0^t \mu_0(s_1)X(s_1) \int_{s_1}^{t} f_1(s_2 - s_1) p_2^{(1)}(s_2, t) ds_2 ds_1 \right].$$

Detection probability and size distribution of adenomas: The probability of detecting an adenoma at age t with the detection threshold y_0 is given by

$$\begin{aligned} P[Y(t) > y_0] &= \sum_{i=y_0+1}^{\infty} \int_0^t \frac{\mu_0(s_1)X(s_1)\int_{s_1}^t f_1(s_2-s_1)P[Y(s_2,t)=i|Y(s_2,s_2)=0]ds_2}{\int_0^t \mu_0(s_1)X(s_1)\int_{s_1}^t f_1(s_2-s_1)ds_2ds_1} ds_1 \\ &= \frac{\int_0^t \mu_0(s_1)X(s_1)p_1^{(AD)}(s_1,t)ds_1}{\int_0^t \mu_0(s_1)X(s_1)\int_{s_1}^t f_1(s_2-s_1)ds_2ds_1}. \end{aligned} \tag{1}$$

And the size distribution of a detectable adenoma at age t is given by

$$\begin{aligned} P[Y(t) = y|Y(t) > y_0] &= \frac{\int_0^t \mu_0(s_1)X(s_1)\int_{s_1}^t f_1(s_2-s_1)P[Y(s_2,t)=y|Y(s_2,s_2)=0]ds_2ds_1}{\int_0^t \mu_0(s_1)X(s_1)\int_{s_1}^t f_1(s_2-s_1)p_2^{(1)}(s_2,t)ds_2ds_1} \\ &= \frac{\int_0^t \mu_0(s_1)X(s_1)\int_{s_1}^t f_1(s_2-s_1)P[Y(s_2,t)=y|Y(s_2,s_2)=0]ds_2ds_1}{\int_0^t \mu_0(s_1)X(s_1)p_1^{(AD)}(s_1,t)ds_1}. \end{aligned} \tag{2}$$

Likelihood for the number and size of detectable adenomas: Following the derivation of L_{11} in the section 'Number and Size Distribution for $K = 1$' in the main text, the likelihood of observing $\{N(t) = n,\ (Y_i(t) = y_i, i = 1, \cdots, n)\}$ is given by

$$\begin{aligned} L_2 \quad \propto \quad & \exp[-\int_0^t \mu_0(s_1)X(s_1)p_1^{(AD)}(s_1,t)ds_1] \times \\ & \prod_{i=1}^{n} \left\{ \int_0^t \mu_0(s_1)X(s_1) \int_{s_1}^t f_1(s_2-s_1)P[Y(s_2,t)=y_i|Y(s_2,s_2)=0]ds_2ds_1 \right\}, \end{aligned}$$

Extension to observations in individuals with no prior CRC. The expressions above can be conditioned on observations in asymptomatic cancer-free individuals as described in the subsection 'PP for both P_1- and P_2-mutations' in the main text. The number of detectable adenomas at time t in cancer-free individual, $N^*(t)$, follows a Poisson distribution with mean $\int_0^t \mu_0(s_1)X(s_1)\int_{s_1}^t f_1(s_2 - s_1)S_2(s_2,t)p_2^{(1*)}(s_2,t)ds_2ds_1$. Therefore, the adenoma prevalence conditioned on no prior CRC is given by

$$1 - \exp\left[-\int_0^t \mu_0(s_1)X(s_1)\int_{s_1}^t f_1(s_2-s_1)S_2(s_2,t)p_2^{(1*)}(s_2,t)ds_2ds_1 \right],$$

where $p_2^{(1*)}(s_2,t) = P[Y(s_2,t) > y_0|Z(s_2,t) = 0, Y(s_2,s_2) = 0]$.

Detection probability and size distribution for adenomas in individuals with no prior CRC: We derive the similar expressions in (1) and (2) conditioned on no prior CRC. The probability of detecting an adenoma at age t with the detection threshold y_0 conditioned on no prior CRC is given by

$$\begin{aligned} P[Y(t) > y_0|Z(t) = 0] &= \sum_{i=y_0+1}^{\infty} \int_0^t \frac{\mu_0(s_1)X(s_1)\int_{s_1}^t f_1(s_2-s_1)S_2(s_2,t)P[Y(s_2,t)=i|Z(s_2,t)=0,Y(s_2,s_2)=0]ds_2}{\int_0^t \mu_0(s_1)X(s_1)\int_{s_1}^t f_1(s_2-s_1)S_2(s_2,t)ds_2ds_1} ds_1 \\ &= \frac{\int_0^t \mu_0(s_1)X(s_1)\int_{s_1}^t f_1(s_2-s_1)S_2(s_2,t)p_2^{(1*)}(s_2,t)ds_2ds_1}{\int_0^t \mu_0(s_1)X(s_1)\int_{s_1}^t f_1(s_2-s_1)S_2(s_2,t)ds_2ds_1}. \end{aligned} \tag{3}$$

And the size distribution of a detectable adenoma at age t conditioned on no prior CRC is as following:

$$\begin{aligned} & P[Y(t)=y|Z(t)=0,Y(t)>y_0] \\ & = \frac{\int_0^t \mu_0(s_1)X(s_1)\int_{s_1}^t f_1(s_2-s_1)S_2(s_2,t)P[Y(s_2,t)=y|Z(s_2,t)=0,Y(s_2,s_2)=0]ds_2ds_1}{\int_0^t \mu_0(s_1)X(s_1)\int_{s_1}^t f_1(s_2-s_1)S_2(s_2,t)p_2^{(1*)}(s_2,t)ds_2ds_1}. \end{aligned} \tag{4}$$

Number and Size Distribution for General K

Here we cover the two special cases: (1) when all pre-initiations are of PP-type, and (2) when they are all of AD-type. Figure 1 in Text S1 illustrates the emerging tree-like structure of case (1). It traces a particular stem cell lineage that leads to a K-stage progenitor cell of an adenoma. The specific notation for this case is introduced below.

PP for all the K pre-initiations

A generalization of the results for $K = 2$ in the main text yields

$$\begin{aligned} N(t) &= \sum_{j=1}^{M(t)} N^{(K-1)}(s_{1j},t) \\ N^{(K-1)}(s_1,t) &= \sum_j N^{(K-2)}(s_1,s_{2j},t) \\ &\vdots \\ N^{(1)}(s_1,\cdots,s_{K-1},t) &= \sum_j N(s_1,\cdots,s_{K-1},s_{Kj},t), \end{aligned} \tag{5}$$

where $N^{(K-1)}(s_{1j},t)$ is the number of detectable adenomas at time t emerging from a P_1-cell born at time s_{1j}, $N^{(K-2)}(s_1,s_{2j},t)$ the number of detectable adenomas at time t emerging from a P_1-cell born at time s_1 and a P_2-cell born at time s_{2j}, and so forth, until we reach $N^{(1)}(s_1,\cdots,s_{K-1},t)$, which is the sum over all the P_K-mutations by time t that occurred at times s_{Kj}'s and which derive from a (random) ancestry of P_l-mutations arising at times s_l, for $l = 1,\cdots,K-1$. As before, $N(s_1,\cdots,s_K,t) = 1$ if the adenoma is detectable at time t and 0 otherwise; that is, $N(s_1,\cdots,s_K,t) = I(Y(s_K,t) > y_0)$. The distribution of this binary random variable is given by

$$p_K^{(1)}(s_K,t) = P[N(s_1,\cdots,s_K,t)=1] = P[Y(s_K,t)>y_0|Y(s_K,s_K)=0], \tag{6}$$

which can be calculated using the formula (1) in the subsection 'Size and detection of adenoma' in the main text for constant parameters.

Given a sequence of $K-1$ ancestral mutations with occurrence times $s_1,\cdots,s_{K-1}$, the occurrences of subsequent P_K-mutations leading to a detectable adenoma at time t follow a non-homogeneous PP with

rate $\mu_{K-1}(s_K)p_K^{(1)}(s_K,t)$, for $s_{K-1} \le s_K \le t$, and $N^{(1)}(s_1,\cdots,s_{K-1},t)$ follows a Poisson distribution with mean $\int_{s_{K-1}}^t \mu_{K-1}(s_K)p_K^{(1)}(s_K,t)ds_K$.

Likelihood for the number and size of detectable adenomas: As before (see the section 'Number and Size Distribution for $K=2$' in the main text), the occurrences of 'special' P_1-mutations, denoted by the process $M^s(t)$, that lead to at least one detectable adenoma at time t, follow a non-homogeneous Poisson process with rate $\mu_0(s_1)X(s_1)p_1^{(K)}(s_1,t)$, for $s_1 \le t$, where $p_1^{(K)}(s_1,t)$ is the probability that a P_1-cell born at time s_1 leads to at least one detectable adenoma at time t, after passing sequentially through $(K-1)$ more pre-initiation stages. Now, given $p_K^{(1)}(s_K,t)$ (see (6)), the probability $p_1^{(K)}(s_1,t)$ can be obtained recursively from

$$\begin{aligned}
p_{K-1}^{(K)}(s_{K-1},t) &= 1-\exp\left[-\int_{s_{K-1}}^t \mu_{K-1}(s_K)p_K^{(1)}(s_K,t)ds_K\right],\\
&\vdots\\
p_2^{(K)}(s_2,t) &= 1-\exp\left[-\int_{s_2}^t \mu_2(s_3)p_3^{(K)}(s_3,t)ds_3\right],\\
p_1^{(K)}(s_1,t) &= 1-\exp\left[-\int_{s_1}^t \mu_1(s_2)p_2^{(K)}(s_2,t)ds_2\right]. \qquad (7)
\end{aligned}$$

Now, let $(i_1,\cdots,i_K)$ be a label enumerating a specific lineage toward a detectable adenoma providing the ancestral information of its entire pathway from the normal stem cell to the founder cell of the adenoma (see Figure 1 in Text S1). Thus, the set of labels $(i_1,\cdots,i_K)$ forms a tree structure branching into n nodes at the top (P_K-level) representing the detectable adenomas with sizes $\{y_i, i=1,\cdots,n\}$. At the bottom (P_1-level), there are m nodes representing the m 'special' P_1-mutations. The i_1th such node has n_{i_1} branches with nodes at the P_2-level, the (i_1,i_2)th node at P_2-level has $n_{i_1i_2}$ branches with nodes at the P_3-level, and so forth. Finally, the $(i_1,\cdots,i_{K-1})$th node at P_{K-1}-level has $n_{i_1\cdots i_{K-1}}$ branches with nodes at P_K-level. Furthermore, let $Y_{i_1\cdots i_K}(t)=y_{i_1\cdots i_K}$ be the size of the $(i_1,\cdots,i_K)$th detectable adenoma. Given the Poisson process assumption for the successive generation of these 'special' mutations, the joint distribution of $\{M^s(t)=m\}$ and the different numbers and sizes of detectable adenomas associated with a specific ancestry of pre-initiations is similar to the formula of $L(m,n_i,y_{ij},j=1,\cdots,n_i,i=1,\cdots,m)$ in the section 'Number and Size Distribution for $K=2$' in the main text, and the form is as following:

$$\begin{aligned}
&e^{-\int_0^t \mu_0(s_1)X(s_1)p_1^{(K)}(s_1,t)ds_1}(m!)^{-1}\prod_{i_1=1}^{m}\left[\int_0^t \mu_0(s_1)X(s_1)e^{-\int_{s_1}^t \mu_1(s_2)p_2^{(K)}(s_2,t)ds_2}\right.\\
&\quad\times(n_{i_1}!)^{-1}\prod_{i_2=1}^{n_{i_1}}\left[\int_{s_1}^t \mu_1(s_2)e^{-\int_{s_2}^t \mu_2(s_3)p_3^{(K)}(s_3,t)ds_3}\right.\\
&\quad\vdots \qquad (8)\\
&\times(n_{i_1\cdots i_{K-1}}!)^{-1}\prod_{i_K=1}^{n_{i_1\cdots i_{K-1}}}\left.\left.\left[\int_{s_{K-1}}^t \mu_{K-1}(s_K)P[Y(s_K,t)=y_{i_1\cdots i_K}|Y(s_K,s_K)=0]ds_K\right]\cdots ds_1\right].
\end{aligned}$$

Note, the typical observation consists of the information at the P_K-level without a tree structure, whereas the likelihood (8) corresponds to the probability of a particular tree leading to the observed P_K-level information. Therefore, the likelihood of observing n detectable adenomas with sizes $y_i, i = 1, \cdots, n$ at age t, i.e,$\{N(t) = n,\ (Y_i(t) = y_i,\ i = 1, \cdots, n)\}$ can be obtained by summing terms like (8) over all possible trees leading to the given P_K-level information. In contrast, when explicit ancestral information is available, then (8) is the relevant likelihood.

Conditioning the relevant expressions on observations from individuals with no prior CRC is straightforward but is not explicitly considered here.

PP for P_1- and AD for all other pre-initiations for General K

The results are similar to those in the subsection 'PP for P_1- and AD for P_2-mutation' above with $f_1(\cdot)$ replaced by the convolution density $(f_1 * \cdots * f_{K-1})(\cdot)$ and the dummy variable s_2 by s_K, the time of P_K-mutation for a normal stem cell. Also, $p_2^{(1)}(s_2, t)$ is to be replaced by $p_K^{(1)}(s_K, t)$ and $Y(s_2, t)$ by $Y(s_K, t)$.

Expected size of detectable adenomas

PP for P_1-mutation for $K = 1$

For the case of PP for P_1-mutation for $K = 1$, the expected size of a detectable adenoma is calculated by

$$E[Y(t)|Y(t) > y_0] = \frac{\int_0^t \mu_0(s_1)X(s_1)p_1^{(1)}(s_1, t)E[Y(s_1, t)|Y(s_1, t) > y_0, Y(s_1, s_1) = 0]ds_1}{\int_0^t \mu_0(s_1)X(s_1)p_1^{(1)}(s_1, t)ds_1}. \tag{9}$$

Now conditioning on no prior CRC, the expected size of a detectable adenoma is as following:

$$\begin{aligned} &E[Y(t)|Z(t) = 0, Y(t) > y_0] \\ &= \frac{\int_0^t \mu_0(s_1)X(s_1)S_2(s_1, t)p_1^{(1*)}(s_1, t)E[Y(s_1, t)|Z(s_1, t) = 0, Y(s_1, t) > y_0, Y(s_1, s_1) = 0]ds_1}{\int_0^t \mu_0(s_1)X(s_1)S_2(s_1, t)p_1^{(1*)}(s_1, t)ds_1}. \end{aligned} \tag{10}$$

For constant parameters, the unconditional and conditional (on no prior CRC) $Y(s_1, t)$ follows a Negative Binomial distribution in the equation (1) and (2) in the main text with $K = 1$.

PP for both P_1- and P_2-mutations for $K = 2$

Similarly, in the case of PP for both P_1- and P_2-mutations for $K = 2$, the expected size of a detectable adenoma is simply given by

$$E[Y(t)|Y(t) > y_0] = \frac{\int_0^t \mu_0(s_1)X(s_1)\int_{s_1}^t \mu_1(s_2)p_2^{(1)}(s_2,t)E[Y(s_2,t)|Y(s_2,t) > y_0, Y(s_2,s_2) = 0]ds_2 ds_1}{\int_0^t \mu_0(s_1)X(s_1)\int_{s_1}^t \mu_1(s_2)p_2^{(1)}(s_2,t)ds_2 ds_1}. \quad (11)$$

And the conditional expected size of a detectable adenoma, given no prior CRC, is given by

$$E[Y(t)|Z(t) = 0, Y(t) > y_0]$$
$$= \frac{\int_0^t \mu_0(s_1)X(s_1)S_3(s_1,t)\int_{s_1}^t \mu_1(s_2)S_2(s_2,t)p_2^{(1*)}(s_2,t)E[Y(s_2,t)|Z(s_2,t) = 0, Y(s_2,t) > y_0, Y(s_2,s_2) = 0]ds_2 ds_1}{\int_0^t \mu_0(s_1)X(s_1)S_3(s_1,t)\int_{s_1}^t \mu_1(s_2)S_2(s_2,t)p_2^{(1*)}(s_2,t)ds_2 ds_1}. \quad (12)$$

For constant parameters, the unconditional and conditional (on no prior CRC) $Y(s_2,t)$ follows a Negative Binomial distribution in the equation (1) and (2) in the main text with $K = 2$.

PP for P_1- and AD for P_2-mutation for $K = 2$

In the case of PP for P_1- and AD for P_2-mutation for $K = 2$, the expected size of a detectable adenoma is calculated by

$$E[Y(t)|Y(t) > y_0] = \frac{\int_0^t \mu_0(s_1)X(s_1)\int_{s_1}^t f_1(s_2 - s_1)p_2^{(1)}(s_2,t)E[Y(s_2,t)|Y(s_2,t) > y_0, Y(s_2,s_2) = 0]ds_2 ds_1}{\int_0^t \mu_0(s_1)X(s_1)p_1^{(AD)}(s_1,t)ds_1}, \quad (13)$$

and the expected size of a detectable adenoma conditioned on no prior CRC is given by

$$E[Y(t)|Z(t) = 0, Y(t) > y_0]$$
$$= \frac{\int_0^t \mu_0(s_1)X(s_1)\int_{s_1}^t f_1(s_2 - s_1)S_2(u_2,t)p_2^{(1*)}(s_2,t)E[Y(s_2,t)|Z(s_2,t) = 0, Y(s_2,t) > y_0, Y(s_2,s_2) = 0]ds_2 ds_1}{\int_0^t \mu_0(s_1)X(s_1)\int_{s_1}^t f_1(s_2 - s_1)S_2(u_2,t)p_2^{(1*)}(s_2,t)ds_2 ds_1}. \quad (14)$$

For constant parameters, the unconditional and conditional (on no prior CRC) $Y(s_2,t)$ follows a Negative Binomial distribution in the equation (1) and (2) in the main text with $K = 2$.

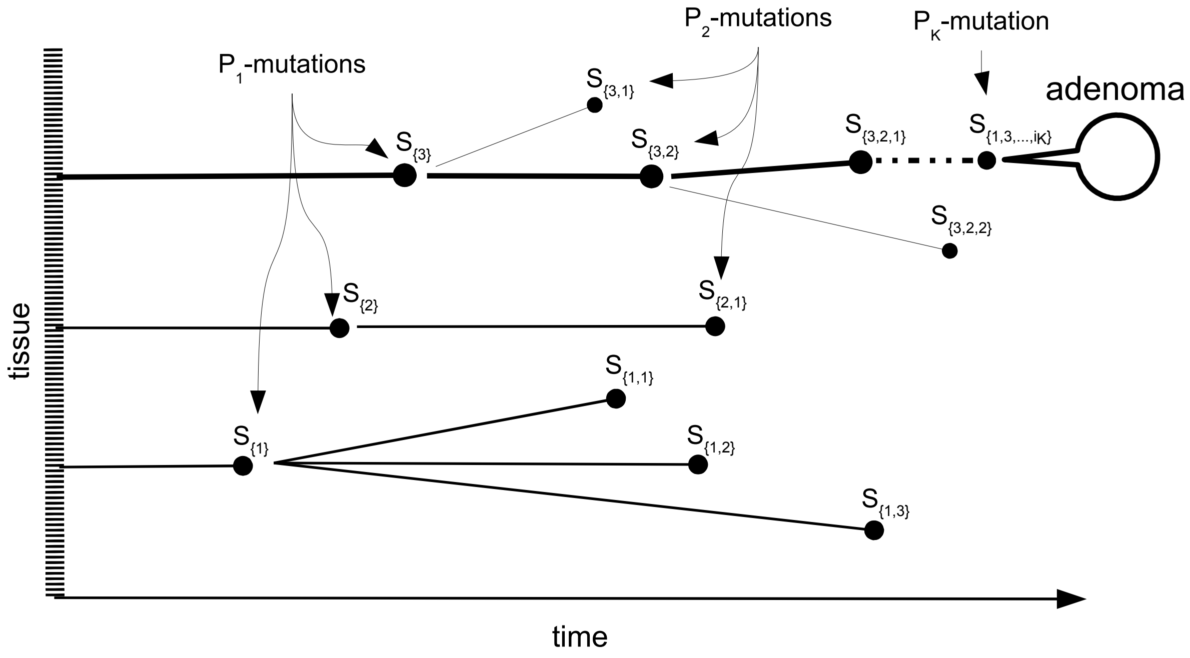

Figure 1. Example PP-type stem cell lineages in adenoma development for the model with general K.

Table 1. Model parameters, notation and terminology.

Symbol	Description	Details
K	Number of pre-initiation stages	We present results for K equal to one, two and general K. Stage $K+1$ represents the adenoma stage in the MSCE model and stage $K+2$ represents the cancer stage
k	pre-initiation stage counter	$k = 1, 2, ..., K$
P_k-mutation	kth pre-initiation event	$k = 1, 2, ..., K$
P_k-cells	The cells which have gone through the P_k-mutation	$k = 0, ..., K+1$. P_0-cells represent the normal stem cells. P_K-cells represent progenitor cells (see below). P_{K+1} represent "initiated cells" (i.e., adenoma cells)
$\mu_k(\cdot)$	Mutation rate of P_k-cells	Rate per year per cell. $k = 0, ..., K$
$\mu_{K+1}(\cdot)$	Malignant conversion rate	Rate at which adenoma cells (P_{K+1}-cells) give rise to a clinical cancer
PP	Poisson Process	The generation of a P_k-cells from a P_{k-1}-cell can be modeled as a non-homogeneous PP with rate $\mu_{k-1}(\cdot)$
AD-type transition	Armitage-Doll transition	The generation of a P_k-cells from a P_{k-1}-cell can be alternatively modeled as an AD-type transition (exponential waiting time with rate $\mu_{k-1}(\cdot)$)
$f_{k-1}(\cdot)$	AD-type transition density function	
X	Number of susceptible normal stem cells	For the colon, X is assumed to be equal to 10^8
$\alpha(\cdot)$	Cell division rate of adenoma stem cells	Per cell per year
$\beta(\cdot)$	Cell death or differentiation rate of adenoma stem cells	Per cell per year
P_K-cell	Progenitor cell of an adenoma	P_K-cells generate initiated cells with rate $\mu_K(\cdot)$. Initiated cells then grow according to a Birth-Death process with rates $\alpha(\cdot)$ and $\beta(\cdot)$. Each progenitor cell generates one adenoma
	Sub-clone	Progenitor cells (P_K-cells) continuously generate new initiated cells. Each new initiated cells generates a sub-clone of adenoma cells independently of the other initiated cells
	Adenoma or adenomatous polyps	Progeny of a progenitor cell (P_K-cell). The total number of stem cells in an adenoma is equal to the sum of cells in the sub-clones originated from a single progenitor cell

Table 1. (Continued)

Symbol	Description	Details
$N(t)$	Number of detectable adenomas	We assume that adenomas are ”detectable” with probability one when their sizes are greater than y_0
$Y_i(t)$	Size of the i-th detectable adenoma	Size is given in number of adenoma stem cells. Detectable means of size greater than y_0
s_k	Time of a P_k-mutation	$k = 1, ..., K$. s_K denotes the arrival time for a progenitor cell, i.e., the onset of the corresponding adenoma
$Y(s_1, ..., s_K, t)$	Size of an adenoma at time t, given that the originating P_1-, P_2-, ..., P_K-mutations occurred at times $s_1, s_2, ..., s_K$, respectively	
$p_n(s_K, t)$	Probability that an adenoma originated at time s_K has size n at time t	
$p_n^*(s_K, t)$	$p_n(t, s_K)$ conditional on not having been previously diagnosed with cancer	
*	In general it applies to quantities computed conditioning on not having been previously diagnosed with cancer	
$p_K^{(1)}(s_K, t)$	Probability that an adenoma is detectable at time t, given that it originated at time s_K	Detectable means of size greater than y_0
$p_k^{(K)}(s_k, t)$	Probability that a P_k-mutation that occurred at time s_k leads to at least one detectable adenoma by time t	
L_K	Likelihood for the number and size of detectable adenomas	K denotes the number of pre-initiation stages in the model
